# Variance in offspring sex ratio and maternal allocation in a highly invasive mammal

**DOI:** 10.1002/ece3.10136

**Published:** 2023-05-25

**Authors:** Sarah M. Chinn, Timothy Smyser, James C. Beasley

**Affiliations:** ^1^ Savannah River Ecology Laboratory, Warnell School of Forestry and Natural Resources University of Georgia Aiken South Carolina USA; ^2^ National Wildlife Research Center, United States Department of Agriculture, Wildlife Services Fort Collins Colorado USA

**Keywords:** fitness, litter size, maternal investment, polytocous, sex allocation theory, Trivers Willard

## Abstract

Skewed sex ratios at birth are widely reported in wild populations, however, the extent to which parents are able to modulate the sex ratio of offspring to maximize their own fitness remains unclear. This is particularly true for highly polytocous species as maximizing fitness may include trade‐offs between sex ratio and the size and number of offspring in litters. In such cases, it may be adaptive for mothers to adjust both the number of offspring per litter and offspring sex to maximize individual fitness. Investigating maternal sex allocation in wild pigs (*Sus scrofa*) under stochastic environmental conditions, we predicted that under favorable conditions, high‐quality mothers (larger and older) would produce male‐biased litters and invest more in producing larger litters with more males. We also predicted sex ratio would vary relative to litter size, with a male‐bias among smaller litters. We found evidence that increasing wild boar ancestry, maternal age and condition, and resource availability may weakly contribute to male‐biased sex ratio, however, unknown factors not measured in this study are assumed to be more influential. High‐quality mothers allocated more resources to litter production, but this relationship was driven by adjustment of litter size, not sex ratio. There was no relationship between sex ratio and litter size. Collectively, our results emphasized that adjustment of litter size appeared to be the primary reproductive characteristic manipulated in wild pigs to increase fitness rather than adjustment of offspring sex ratio.

## Graphical abstract

Skewed sex ratios at birth in highly polytocous species are complicated since maximizing fitness may include trade‐offs between sex ratio and the size and number of offspring in litters. We investigated the offspring sex ratio and maternal allocation in wild pigs (*Sus scrofa*) in relation to maternal quality and stochastic environmental conditions. Maternal genetics, age, and body condition may weakly skew the sex ratio toward males and allocation to litter production, but adjustment of litter size appeared to be the primary reproductive characteristic manipulated to increase maternal fitness rather than adjustment of offspring sex ratio.

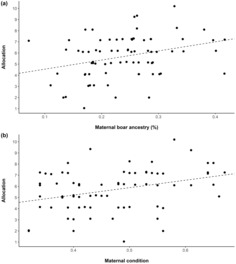

## 1 INTRODUCTION

Sex allocation theory (Charnov, 1982; Frank, 1990), particularly sex ratio variation (i.e., the proportion of male and female offspring produced), is a widely addressed concept in life‐history theory (Charnov, 1982; Clutton‐Brock & Iason, 1986; Fisher, 1930; Frank, 1990). Although previous studies have documented skewed sex ratios favoring either males or females at birth, the question of whether modulation of offspring sex is an adaptive strategy as a means for parents to increase their own fitness remains unclear, particularly for vertebrates (for reviews, see Clutton‐Brock & Iason, 1986; Hardy, 1997; Williams, 1979). Recent studies on vertebrate species that employ either environmental (e.g., reptiles) or gonadal sex determination (birds and mammals) suggest mechanisms responsible for sex ratio variation are influenced by individual parental phenotype (i.e., maternal condition), genotype (i.e., genetic pathways and epigenetics) and physiology (i.e., corticosteroids), social changes (i.e., status and interactions), population density, and stochastic environmental conditions (Bowden et al., 2000; Kruuk et al., 1999; Navara, 2013).

The influence of parental conditions on reproductive success (Festa‐Bianchet et al., 1998; Stearns, 1992) is one of the most commonly tested predictors of offspring sex ratio (Trivers & Willard, 1973). The Trivers Willard Model (TWM) predicts, in polygynous species in which males have greater variance in individual fitness, mothers in good body condition or with access to quality resources should invest more in sons provided that: sons benefit more than daughters from extra allocation of resources, offspring quality correlates to adult quality, and offspring quality is a good indicator of maternal quality. Thus, females should be able to modulate their offspring sex ratio in response to factors that could modify both their own lifetime reproductive success and the reproductive success of their progeny. Though well studied in vertebrate species that produce a single offspring (Clutton‐Brock et al., 1984; Hewison & Gaillard, 1999; Sheldon & West, 2004), it is not well defined if or how the TWM applies to polytocous species (i.e., those that produce several offspring per litter) because the trade‐offs between body size and number of offspring must also be considered (Myers, 1978; Smith & Fretwell, 1974; Williams, 1979).

For polygynous polytocous species, it may be adaptive for the mother to manipulate both the number of offspring per litter and their sex to maximize the reproductive returns of her offspring, according to her own available resources (Williams, 1979). Fisher (1930) predicted that parental expenditure in both sexes should be equal at the end of parental care because males typically have higher preweaning mortality compared with females, and if the sex ratio at birth is male‐biased, it tends to balance out by the end of the offspring dependency period. However, males are larger at birth and develop at a faster rate both in utero and during the postparturition investment period in most mammals (Anderson & Fedak, 1987; Mittwoch, 1993). Thus, for mammals, it can be assumed that there is a differential cost for producing male and female offspring (Clutton‐Brock et al., 1981). As the number of offspring within a litter increases, reproductive returns from allocating more resources to male and female offspring will become equivalent and the mother may increase her fitness by adjusting litter size rather than offspring sex ratio (Frank, 1990). Thus, predictions of the TWM are complicated (Myers, 1978; Williams, 1979). In addition to accounting for multiple offspring, the Williams Model considers the effects of resource availability that further complicate testing sex ratio theory, as the resources available to the mother can modulate allocation in both litter size and sex ratio (Williams, 1979). At one extreme, mothers with limited resources should have smaller and female‐biased litters, while at the other extreme, mothers with ample resources should have large and male‐biased litters. Mothers should be selected to have intermediate‐sized litters with approximately equal number of males and females under moderate resource availability (Williams, 1979). Thus, if offspring sex ratio adjustment is an adaptive strategy, it is reasonable to expect the interaction between environmental factors (e.g., extreme climatic events) and individual phenotype (e.g., age) should predictably influence the offspring sex ratio. Studies of sex allocation exist in the literature for avian species, where parental care is typically provided by both parents (Clutton‐Brock, 1986; Dijkstra et al., 1990; Ellegren et al., 1996) and for small mammals, especially in a laboratory setting (Cameron et al., 2008; Pratt & Lisk, 1989). However, neither the TWM (Schindler et al., 2015, but see Sidorovich et al., 2007) nor the Williams model that evaluates the interaction between both environmental drivers (abiotic) and individual attributes (biotic) on sex ratio (but see Stewart et al., 2005; Servanty et al., 2007) have been thoroughly evaluated among large, wild mammalian species with multi‐offspring litters.

**FIGURE 1:**
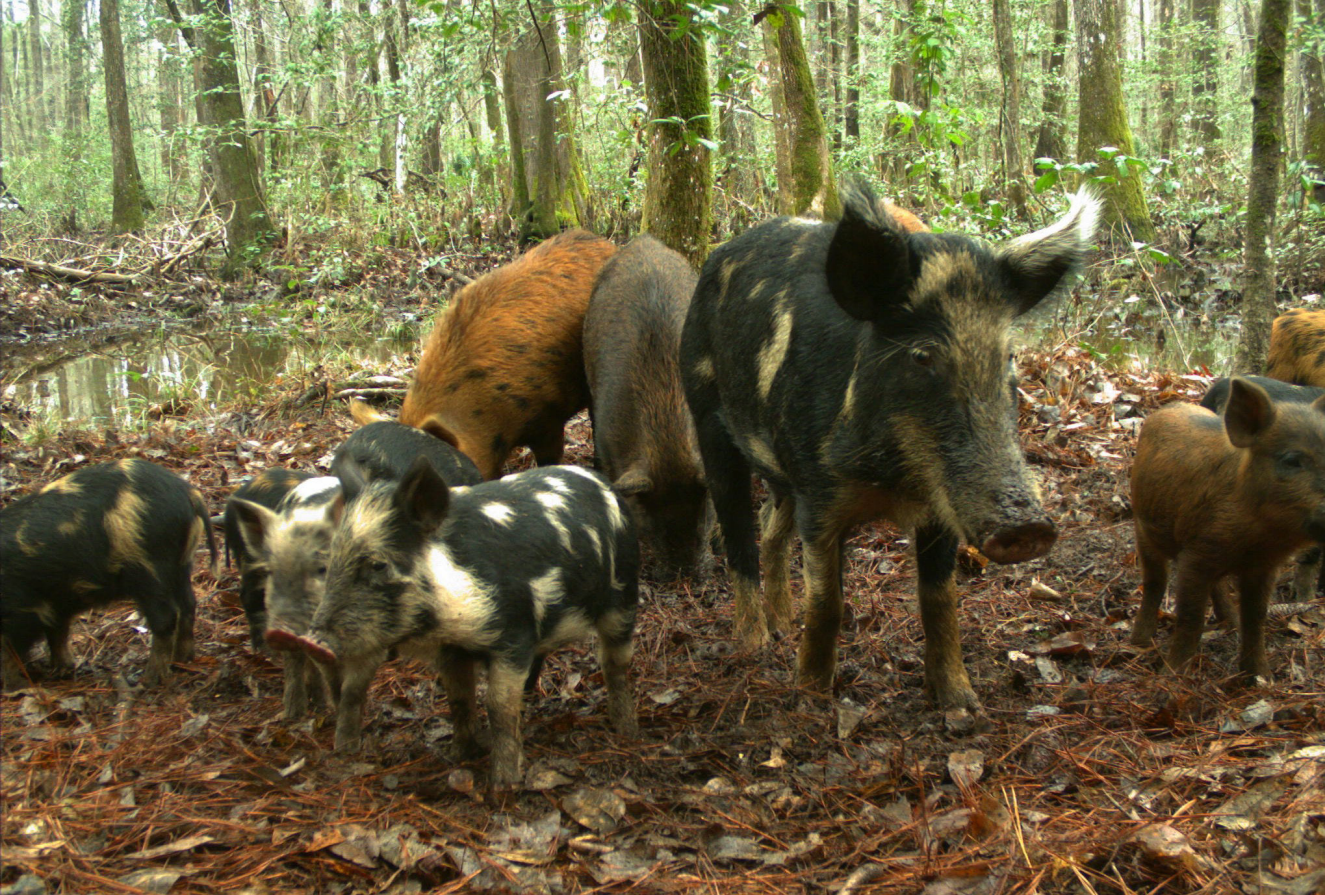
Wild pig (*Sus scrofa*) mature female, juveniles, and piglets on the Savannah River Site, Aiken, SC, USA.

Invasive wild pigs (*Sus scrofa*) are an ideal species in which to study sex allocation theory and particularly offspring sex ratio (Figure 1). If maternal quality and environmental conditions bias maternal allocation to the sex with the greater variation in reproductive potential, then the reproductive potential of the offspring can be influenced by maternal care. And, there can be higher reproductive returns when the trade‐off between fetal sex modulation and the number of offspring is taken into account. Wild pigs exhibit a polygynous mating strategy, are highly polytocous with a mean fetal litter size of 5.3 in North America (Snow et al., 2020), and have the highest reproductive rate compared with any other mammal of similar size (Taylor et al., 1998). Established throughout much of the contiguous United States, contemporary populations of wild pigs represent a hybrid swarm with animals of mixed wild boar and domestic/feral pig ancestry (Keiter et al., 2016; Smyser et al., 2020). Relative genetic influences from wild boar versus domestic/feral pig vary within and among populations, which may have ramifications for litter size given that domestic pigs have been under artificial selective pressures to, in part, maximize reproductive output (Taylor et al., 1998). Studies have documented increased litter size and earlier sexual maturity of wild boar in their native range (Briedermann, 1986; Mauget, 1982; Servanty et al., 2009) and wild pigs in North America (Baber & Coblentz, 1986; Chinn et al., 2022; Johnson et al., 1982; Matschke, 1964) in response to increased mast availability as well as to pulses in other seasonal crops (wild boar: Massei et al., 1996; Frauendorf et al., 2016; wild pig: Barrett, 1978; Warren & Ford, 1997). Abundant resources may increase maternal condition and in turn, females in superior condition may have the physiologic resources to increase offspring size, number, and adjust sex ratio as a means to increase lifetime reproductive success.

Using reproductive data collected over multiple years with varying resource availability, we applied broad sex allocation theory, specifically in terms of a suite of biotic and abiotic factors that have been proposed to drive adaptive adjustment of fetal sex ratio and parental allocation, to test the applicability of the TWM and Williams Models to a highly polytocous large mammal. First, we hypothesized maternal phenotype would influence fetal sex ratio, a test of the TWM. If supported, we predicted older and larger females would produce male‐biased litters. Second, we hypothesized environmental conditions would influence fetal sex ratio and the interaction of these abiotic conditions and maternal attributes would increase the magnitude of any effects on fetal sex ratio, by testing if food resources affected sex ratio as predicted by the TWM. We predicted that in years with abundant resources such as pulsed food resources from oak (*Quercus* spp.) mast, females would be in better nutritional condition (i.e., more fat reserves) and produce male‐biased litters. Third, to test the Williams Model, we hypothesized maternal ancestry (i.e., ancestral genomic contributions from wild boar vs domestic pig), age, body condition, and environmental factors would influence the relative allocation in litter production, a cost metric reflecting litter size and offspring sex ratio (Williams, 1979). We predicted increased allocation toward larger litters and more males from females that were older, larger, and in better nutritional condition from high food availability. Given a polygynous breeding strategy and female‐dominated social groups, we predicted mothers with greater wild boar ancestry would produce male‐biased litters. Finally, we expected sex ratio would vary in relation to litter size, with smaller litters being male‐biased and larger litters being female‐biased, a trade‐off in the Williams Model.

## 2 MATERIALS AND METHODS

### 2.1 Study area

This study was conducted at the Savannah River Site (SRS), a 78,000 ha U.S. Department of Energy facility located in Aiken, Barnwell, and Allendale counties, South Carolina, USA. Habitat on the SRS is mostly comprised of upland pine forests, bottomland hardwood forests, and swamps. Wild pigs on the SRS are descendants of feralized domestic pigs that were not recovered by farmers after the government purchased the land in 1950 (Mayer, Beasley, et al., 2020). A subsequent introduction of wild boar hybrids occurred on the SRS in the 1980s, which have since expanded throughout the site and hybridized with established feral domestic pigs. The contemporary wild pig population represents hybrids of Western heritage breeds of domestic pigs and wild boar with considerable variation in wild boar (6–60%) versus heritage breed ancestry among individuals (Chinn et al., 2022; Smyser et al., 2020). Despite being lethally managed since 1956, the wild pig population on the SRS has continued to increase over the last several decades (Keiter et al., 2017; Mayer, Beasley, et al., 2020). Wild pigs are abundant throughout the entire SRS (Mayer, Beasley, et al., 2020), with an estimated population size of >5000 individuals at the time of this study (Keiter et al., 2017).

### 2.2 Data collection

We sampled from live‐trapped (and consequently released for other studies, e.g., neonate survival (Chinn et al., 2021)) and humanely euthanized wild pigs throughout the year between March 2017 and July 2019 according to established protocols (A2015 12‐017). Wild pigs were primarily sampled from ongoing wild pig management on the SRS, although additional individuals were culled as part of other research activities. In wild pigs, age at first reproduction is typically driven by a minimum body size (~30 kg, which may be dependent on available forage) such that juveniles <6 months old may be sexually mature (Chinn et al., 2022; Comer & Mayer, 2009). We collected fetal data, tissue for genetic analysis, and morphological measurements from females >27 kg to quantify litter and maternal attributes (*n* = 160). We weighed and measured each female dorsally from the snout to the base of the tail. We calculated a standardized body condition index (subsequently referred to as maternal condition) for each individual as mass/length (LaBocha et al., 2014). Our study encompassed years of differential mast availability, with 2017 as high availability and 2018 and 2019 as having low to moderate mast availability (J.C. Beasley, personal observation). We measured extraneous fat reserves (i.e., rump fat between the ischium and ilium bones, ~3 cm lateral from the spine), a quantitative measurement of maternal nutritional condition, from culled females and used it as a proxy for local resource availability. We assessed age by tooth eruption and replacement patterns (Matschke, 1967; Mayer, 2002), and individuals were classified into 3 age classes: juvenile (between 6 months and 1 year), yearling (>1–1.5 years), and adults (>1.5 years old). Juveniles, as young as 3–5 months of age (Comer & Mayer, 2009), and yearlings can be pregnant because wild pig age classes are based on morphological characteristics (size, mass, tooth eruption) and not sexual maturity. Because we limited our sampling to females >27 kg, we may have missed some young, pregnant individuals for this study. If present, fetuses were removed, weighed, and measured in a straight line from crown to rump (CRL). The average gestation is 115 days and fetuses were aged based on CRL (Henry, 1968). Fetuses ≥36 days old were sufficiently developed to visually determine sex.

We quantified the ancestry of wild pigs following methods described by Smyser et al. (2020). In summary, we extracted DNA from tissue using a magnetic bead‐based extraction protocol (MagMax DNA; Thermo Fisher Scientific) and genotyped animals with Genomic Profiler for Porcine HD (GeneSeek; a Neogen Corporation; Ramos et al., 2009) with Illumina BeadChip microarray chemistry. This provided 29,375 biallelic single nucleotide polymorphisms (SNP), from which we estimated the ancestry of individual wild pigs using ADMIXTURE version 1.3.0 (Alexander et al., 2009) to query individual wild pig genotypes against a comprehensive reference set for *Sus scrofa* (2516 genotypes sampled from 105 domestic breeds, 23 wild boar populations in their native range, and 4 sister taxa) organized into 17 genetically cohesive ancestry groups (Smyser et al., 2020). The analysis resulted in the proportionate association of individual wild pig genomes among the 17 ancestry groups representing the *Sus scrofa* wild‐domestic species complex with individual ancestry associations summing to 1. Given that association with wild boar and heritage breeds represented the vast majority of the ancestral composition of the SRS population, we included only wild boar ancestry as a variable in subsequent analyses.

### 2.3 Data analysis

To confirm rump fat was a suitable proxy for resource availability, we tested whether increased acorn mast production in 2017 resulted in greater rump fat depth compared with the other years of our study using an Analysis of Variance (ANOVA) test. We calculated the sex ratio for each litter as the proportion of males. We tested if the sex ratio differed significantly from parity using a one‐sample *t*‐test (i.e., the null hypothesis, *μ* = 0.5).

#### 2.3.1 Trivers Willard model

We used the *lme4* package (Bates et al., 2015) in R (R Core Team, 2021) to fit logistic regression models with binomial distribution with the proportion of males as the response variable to test the influence of a suite of maternal and environmental attributes on fetal sex ratio (Wilson & Hardy, 2002). We included maternal age class, maternal condition, resource availability (i.e., rump fat, a measure of environmental variation on food availability), litter size, percent wild boar ancestry, and select interactions. Age and litter size within our study site have been shown to be correlated (Chinn et al., 2022), therefore, we did not include both variables within the same model. We tested if maternal age and condition were correlated by performing an ANOVA. We created an a priori candidate set of biologically relevant models using variables that were not highly correlated. With preliminary analysis, we found that complex models (e.g., multiple interactions) did not perform well, thus we limited our candidate models to 3 fixed variables (e.g., 2 fixed effects and 1 interaction). We used Akaike's Information Criterion (AIC) corrected for small sample sizes (AICc) and model weights to determine which candidate models provided the best support for the data. Models within ΔAICc ≤2 of the top model were considered influential and reported. We used model weight to evaluate the strength of influence among competing models (Burnham & Anderson, 2002).

#### 2.3.2 Williams model

Since the time of gestation differed among sampled pregnant females (i.e., individuals were in different stages of pregnancy), we used the proportional difference in mass between male and female fetuses within each litter to quantify the relative cost of producing a male or female fetus (for similar methods see Fernández‐Llario et al., 1999; Servanty et al., 2007). Overall, male fetuses were 2% heavier than females, thus the direct cost of producing a male was slightly greater compared to producing a female (i.e., on average 1 male = 1.02 female). Using these averages, we calculated the maternal allocation for a litter, a function of the number of fetuses and their sex, to determine whether there was a trade‐off between litter size and offspring sex ratio. For example, a litter with 2 females and 2 males would require an allocation of 2 × 1 + 2 × 1.02 = 4.04 units, whereas a litter with 4 females or 4 males would require an allocation of 4.00 and 4.08 units, respectively. To further justify our methods, we calculated the average fetal mass of the males and females within each litter and plotted the difference in mass (e.g., average male fetus mass – average female fetus mass) over the gestation period (day 36–115). Since fetuses were of differing ages, we assessed if maternal allocation was constant through gestation and that our metric for maternal allocation (see above) was not just applicable to the latter portion of gestation. We calculated the differences in average mass of male fetuses compared to female fetuses across the gestation period to determine if there was a bias in allocation (i.e., mass) toward one sex (e.g., Fernández‐Llario et al., 1999; Servanty et al., 2007). Further, we used linear models to test if fetus growth (using mass) varied between male and female fetuses and if there was sex‐biased mortality along the gestation period by assessing any changes in sex ratio during the gestation period. We also tested if maternal attributes and food availability influenced allocation to litter production. Using the same criteria as above for AICc model selection, we used linear models to determine if maternal ancestry (percent wild boar), maternal age class, maternal condition, resource availability (rump fat), and selected interactions affected allocation to fetuses. In a post‐hoc analysis, we used a linear model to test if litter size and sex ratio were related to maternal condition to determine if either litter size or sex ratio were more influential in driving allocation during gestation. Finally, we calculated the mean sex ratio for all observed litter sizes to test if sex ratio varied in relation to litter size, specifically to determine if smaller litters were male‐biased and larger litters were female‐biased.

We centered and z‐transformed continuous variables to a mean of 0 and a standard deviation of 1 to allow for standardized comparison for all analyses. Variance inflation factors (VIF) were <3, indicating no multicollinearity between continuous fixed effects. We checked for overdispersion and that the standardized residuals were randomly distributed around zero with respect to the fitted values (Wilson & Hardy, 2002). We assessed model residuals for normality. All analyses were performed in R (version 4.0.5).

## 3 RESULTS

Rump fat deposits were trending larger in 2017 (*n* = 22, $\bar{X}$ ± SE = 1.52 ± 0.20 cm), a high mast production year, compared with 2018 and 2019 (*n* = 43, 1.21 ± 0.10 cm and *n* = 19, 1.30 ± 0.17 cm, respectively), low to moderate mast production years. Although the difference in rump fat deposits between years was not statistically different for the pregnant females used in the proceeding sex ratio analyses (*F*_2,128_ = 2.51, *p* = .09), there was a significant difference in rump fat between years when all reproductively mature females sampled between 2017 and 2019 were analyzed such that females in 2017 had significantly more rump fat compared to 2018 or 2019 (*n* = 449, *F*_1,448_ = 8.01, *p* = .006; 2017 = 1.00 ± 0.05; 2018 = 0.75 ± 0.04; 2019 = 0.82 ± 0.07). Thus, we believed that rump fat was a suitable proxy for resource availability for wild pigs on the SRS during our study period. Of the 160 pregnant females, fetal litter size ranged from 1 to 12, (5.43 ± 0.14, mode = 6), and 89 females had fetuses that were old enough to visually determine sex (range: 1–11, 5.72 ± 0.21, mode = 6). However, 13 litters were excluded from the logistic regression analysis because we did not have the complete set of morphometric variables (i.e., missing maternal condition or fetal measurements). Litters within our population were male‐biased, with an average sex ratio across all litters (*n* = 89) of 0.55 (significantly different from parity; *t*_88_ = 2.19, *p* = .03).

### 3.1 Trivers‐Willard model

Maternal age and condition were correlated; therefore, we did not include both variables in the same candidate model. Our analysis evaluating whether fetal sex ratio was influenced by maternal and environmental attributes resulted in seven competitive models (Table 1). The most influential model included the variables of wild boar ancestry (*β* = 0.13), maternal condition (*β* = −0.09), and their interaction (*β* = −0.22). However, the null model ranked within Δ2 AICc of this model, therefore, none of our predicted explanatory parameters (ancestry, maternal age class, maternal condition, litter size, or resource availability) influenced the sex ratio (Table 1). The other candidate models within Δ2 AICc included maternal age (*β*_yearling_ = 0.07, *β*_juvenile_ = −0.53), percent wild boar ancestry, maternal condition, resource availability (*β* = 0.09), and maternal age + resource availability, all of which had low model weights (Table 1). Thus, none of our measured parameters provided support for the TWM.

**TABLE 1:** Generalized linear model results (within ΔAICc ≤2 of the top model) evaluating the influence of maternal attributes and environmental variables on wild pig (*Sus scrofa*) sex ratio at the Savannah River Site, Aiken, SC, USA.

Model	df	AICc	Delta	Weight
logit(sex ratio) = boar + condition + boar*condition	4	234.89	0	0.13
logit(sex ratio) = null model	1	234.99	0.1	0.13
logit(sex ratio) = age	3	235.66	0.76	0.09
logit(sex ratio) = boar	2	236.39	1.5	0.06
logit(sex ratio) = condition	2	236.4	1.51	0.06
logit(sex ratio) = fat	2	236.46	1.57	0.06
logit(sex ratio) = age + fat	4	236.68	1.78	0.05

*Note*: Age, maternal age; boar, percent of wild boar ancestry; Condition, maternal body condition index (mass/length); Null, no covariates.

### 3.2 Williams model

**FIGURE 2:**
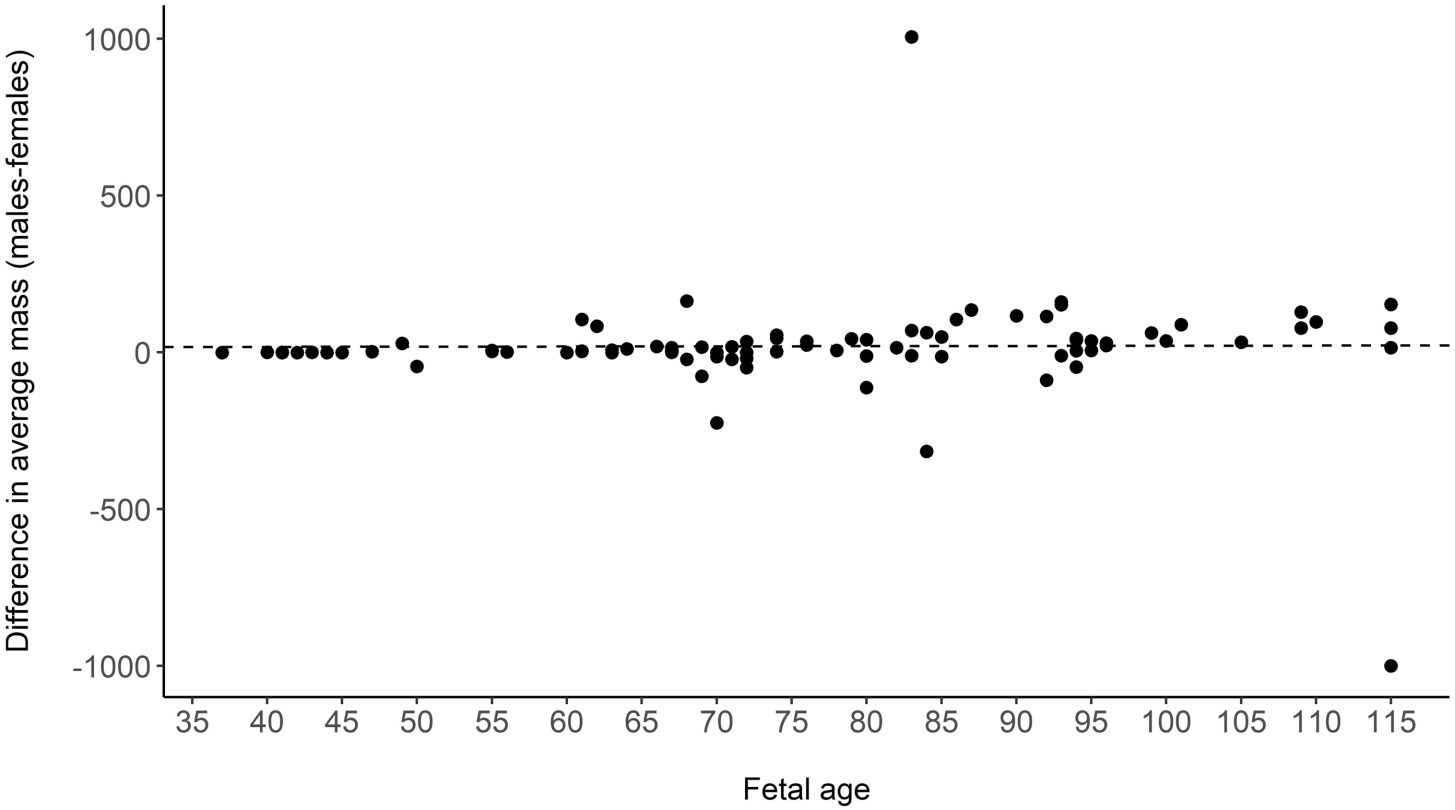
Average fetal mass (average male – average female) for each litter (*n* = 89) across the gestation period (fetal age 36–115 days, when sex was determined visually) for wild pigs (*Sus scrofa*) at the Savannah River Site, Aiken, SC, USA from 2017 to 2019. The dotted line denotes the fitted regression line.

**FIGURE 3:**
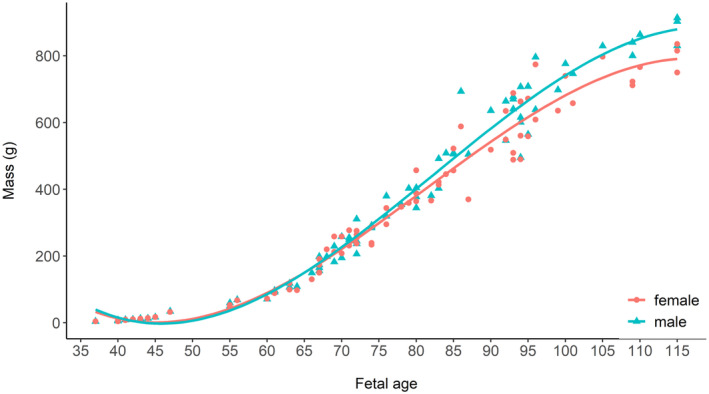
Fetal mass for males and females and the smoothed regression line (third order polynomial) through gestation (fetal age 36–115 days, when fetal sex was determined visually) for wild pigs (*Sus scrofa*) at the Savannah River Site, Aiken, SC, USA from 2017 to 2019.

**FIGURE 4:**
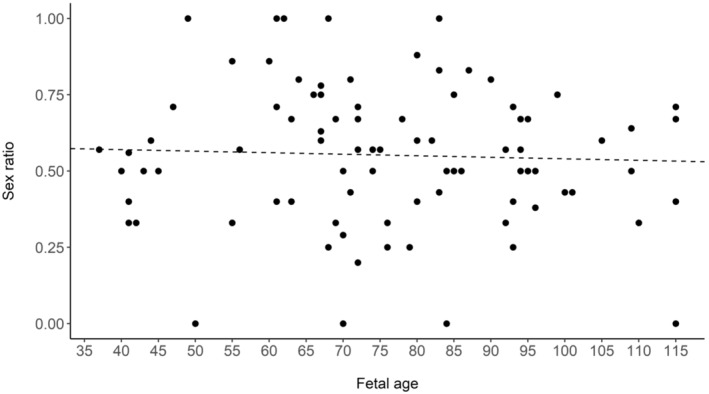
Sex ratio for each litter (*n* = 89) across gestation (fetal age 36–115 days) for wild pigs (*Sus scrofa*) at the Savannah River Site, Aiken, SC, USA from 2017 to 2019. The dotted line denotes the fitted regression line.

The average mass of male and female fetuses was 350.82 ± 280.03 g ($\bar{X}$) and 345.11 ± 261.16 g, respectively. However, mass was not significantly different between sexes (*p* = .82). We found no difference between the average fetal mass of males and females through gestation (Figure 2; *F*_1,85_ = 0.004, *p* = .92, *R* ^2^ = −0.01). Although mass did not statistically differ between males and females, males were on average 2% heavier compared with females, and male and female fetuses grew similarly through gestation until ~75 days old after which males grew faster (Figure 3; *T* = 3.24, *p* = .001). Therefore, males appeared to require differential allocation by mothers, especially at the end of gestation. This is likely an additive cost and thus potentially biologically relevant because wild pigs produce multiple offspring of each sex per litter. Fetal sex ratio did not change through gestation, so our sample did not support sex‐biased fetal mortality after day 37 (Figure 4; *F*_1,87_ = 0.21, *p* = .65, *R* ^2^ = −0.009).

**FIGURE 5:**
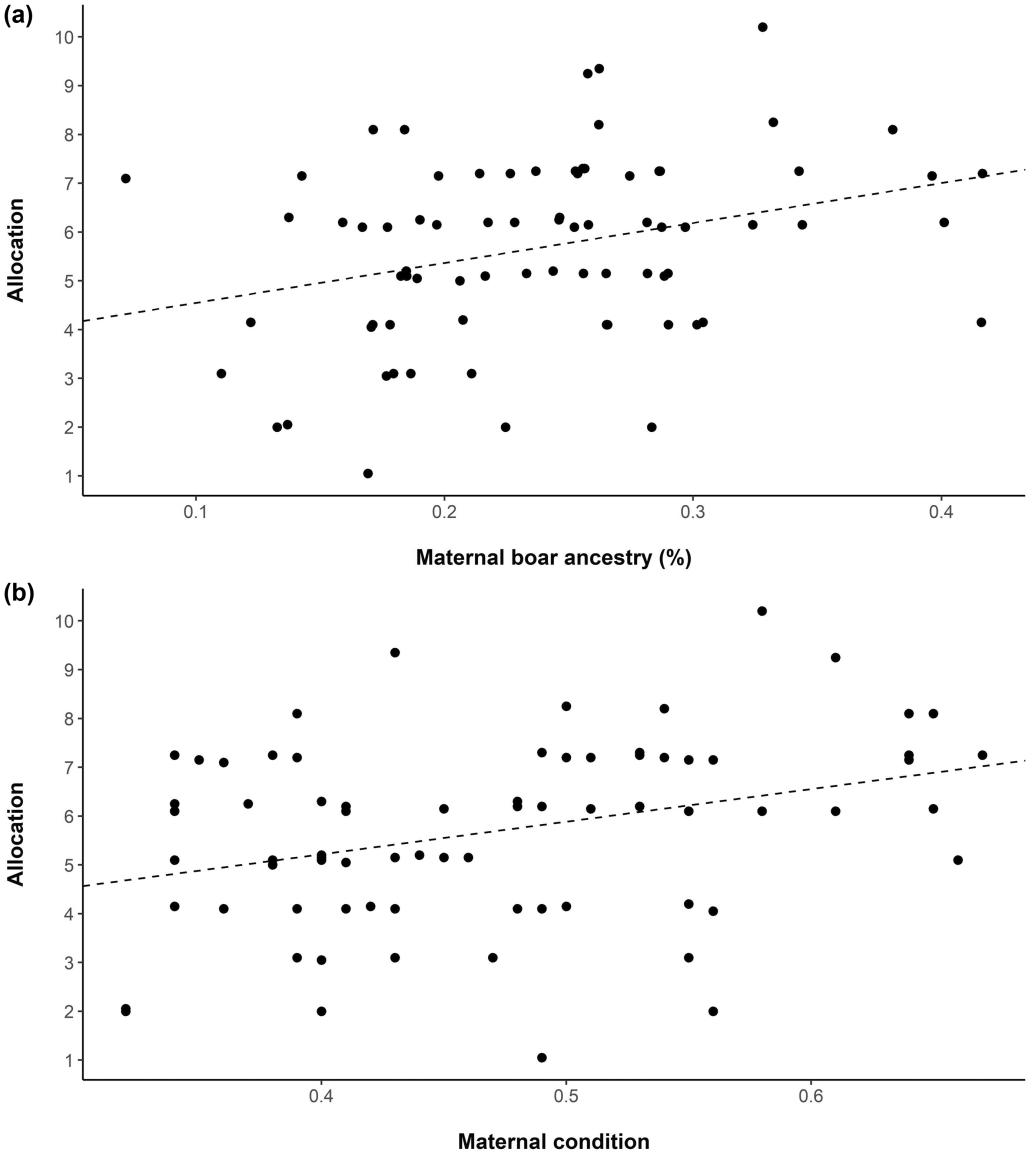
Average maternal allocation (calculated as the sum of the cost of each offspring according to sex) toward litter production in relation to (a) maternal ancestry (percent wild boar) and (b) maternal condition for wild pigs (*Sus scrofa*) at the Savannah River Site, Aiken, SC, USA from 2017 to 2019.

Model selection results pertaining to allocation in litter production identified 2 competing models. Percent of wild boar ancestry and maternal condition were present in both top‐performing models, indicating some support for Williams' hypothesis that the mother's attributes influenced her allocation toward producing a larger litter with many males (Table 2). The top‐ranked model included the percent of wild boar ancestry (*β* = 0.47) maternal condition (*β* = 0.51) and the second‐ranked model included the interaction term (*β* = 0.12). Allocation to litter size increased as boar ancestry increased (*F*_1,74_ = 8.31, *p* = .005, *R* ^2^ = 0.09; Figure 5a) and as maternal condition increased (*F*_1,74_ = 9.40, *p* = .003, *R* ^2^ = 0.10, Figure 5b).

**TABLE 2:** Linear model results (within ΔAICc ≤2 of the top model) evaluating the influence of maternal and environmental attributes on wild pig (*Sus scrofa*) maternal allocation in litter production at the Savannah River Site, Aiken, SC, USA.

Model	Df	AICc	Delta	Weight
Allocation = boar + condition	4	299.7	0	0.36
Allocation = boar + condition + boar*condition	5	301.33	1.62	0.16

*Note*: Boar, percent of wild boar ancestry; Condition, maternal body condition index (mass/length).

**FIGURE 6:**
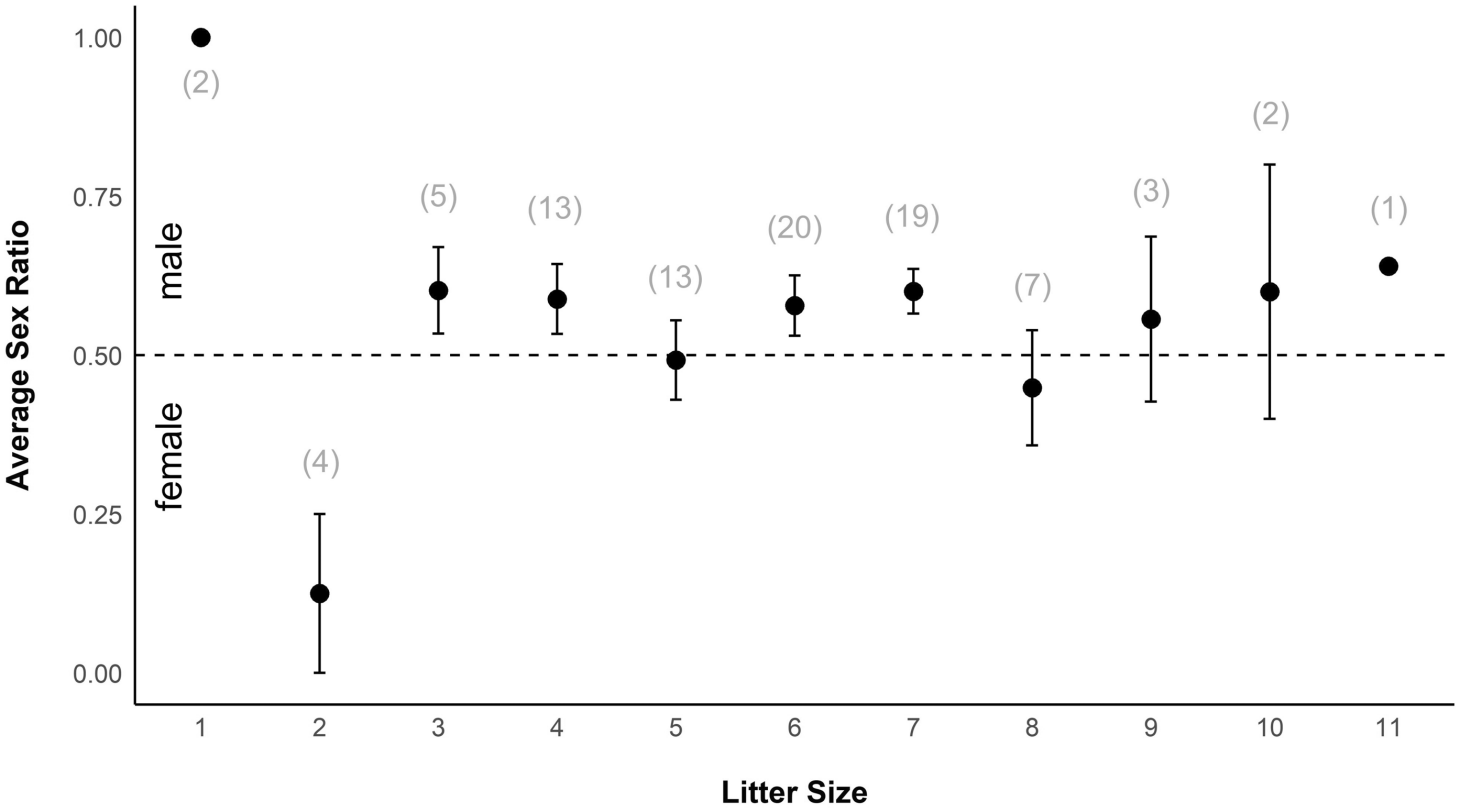
Average sex ratio (mean proportion of males) ± SE for a given litter size for wild pigs (*Sus scrofa*) at the Savannah River Site, Aiken, SC, USA from 2017 to 2019. The dotted line denotes even sex ratio (equal number of males and females) and the number in parentheses represents the number of litters.

While parental allocation was influenced by maternal condition, we found that maternal condition was positively related to litter size (*t* = 3.12, *p* = .003) but not to sex ratio (*p* = .71). The observed relationship between allocation and maternal condition was thus driven by litter size rather than sex ratio. Contrary to a similar study on wild boar sex ratio in their native range (Servanty et al., 2007), we did not observe a relationship between litter size and sex ratio (Figure 6).

## 4 DISCUSSION

We tested sex ratio and maternal allocation theory in relation to a suite of biotic and abiotic factors on a globally distributed and highly polytocous large mammal. The TWM predicts that to maximize their own reproductive success, high‐quality mothers should produce more sons, if males have higher individual fitness. Tests of the TWM are complicated for polytocous species because maternal resources must be spread across multiple offspring. Therefore, the number of offspring and the relative size difference (if any) between the sexes is important for determining the allocation of resources toward the offspring sex ratio (Williams, 1979). In our study, female wild pigs produced litters with sex ratios that differed significantly from parity. None of our measured biotic or abiotic attributes substantially contributed to sex ratio variation among litters, suggesting female wild pigs may employ alternative strategies for maximizing their fitness. Indeed, we found that higher‐quality mothers produced more offspring, supporting the Williams Model. Collectively, our results emphasized that in polytocous species like the wild pig, preferential selection for adjustment of litter size appears to be the primary reproductive characteristic manipulated to increase reproductive success and concomitantly fitness rather than adjustment of offspring sex ratio. Production of the largest litter size possible in which all offspring survive may provide the greatest reproductive returns, even if the fetal sex ratio is female‐biased.

Bearing multiple offspring introduces a fitness trade‐off between the number of offspring that can be produced and their sex, if there is a differential cost between the sexes (Myers, 1978; Smith & Fretwell, 1974; Williams, 1979). While sex ratios become more female‐biased with increasing litter size in older domestic piglets (Gorecki, 2003), the relationship between litter size and fetal sex ratio in free‐ranging wild boar and wild pigs is less clear. Servanty et al. (2007) found that small wild boar litters tended to be male‐biased, whereas larger litters were female‐biased. They suggested females with larger litters might be limited in the additional allocation required for males because males were larger and therefore more costly to produce compared with females. While the authors reported sex ratio was male‐biased in small litters and female‐biased in large litters, the 95% confidence intervals in their analysis included equal sex ratio implying that there was no statistical difference in sex ratios from parity for any litter size, except for a litter size of 4 individuals (see figure 2 in Servanty et al., 2007). Similarly, Fernández‐Llario et al. (1999) found no relationship between sex ratio and litter size in wild boars in Spain. While no significant relationship was apparent in our study, intermediate litter sizes (*n* = 3, 4, 6, 7) had a higher proportion of males. A recent study of wild boars in South Korea found that the heaviest fetuses were male‐biased, suggesting this was a strategy to increase maternal fitness (Lee, 2022). If the average litter size of wild pigs is adapted to the largest number of offspring a female can successfully provision, producing more males within litters of optimal size (i.e., the litter size where offspring survival is highest) could be selected to maximize reproductive returns and thus fitness. Expansion upon this notion by increasing sample sizes and sampling from different wild pig populations could provide more insight into this potential mechanism for optimizing the inclusive fitness of a widely successful invasive species.

Percent wild boar ancestry may have weak influence on the variance of fetal sex ratio. Within the contiguous U.S., populations of invasive wild pigs overwhelming represent the hybrid descendants of feralized domestic pigs and introduced wild boar (Keiter et al., 2016; Smyser et al., 2020), with local variation in ancestral origins based on geography and the history of wild boar releases. On average, wild pigs have larger litters ($\bar{X}$= 5.3 piglets in North America (Snow et al., 2020); 5.7 piglets in this study) than wild boar (4–5 piglets, Comer & Mayer, 2009), but smaller litters compared with domestic pigs that range from an average of 9–12 piglets (Alfonso, 2005; Asdell, 1964; Gorecki, 2003; Hagen & Kephart, 1980). Given the origins of wild pigs include domestic stock that were selectively bred to favor high reproductive rates, early sexual maturity, and large litter sizes (Snow et al., 2020; Taylor et al., 1998), there may be differences in reproductive ecology among populations due to differences in the ancestral composition of populations (Smyser et al., 2020). We predicted that females with greater wild boar ancestry would have male‐biased litters because *Sus scrofa* exhibits a promiscuous breeding strategy and social groups are dominated by females. Males disperse from the family group and have greater reproductive potential compared to daughters that stay within the sounder. However, this could be dependent on the proportion of wild boar ancestry within each local population of wild pigs, which is not currently well known. Although all individuals evaluated within the SRS population represent Western heritage‐wild boar hybrids, individuals vary considerably in ancestry (Chinn et al., 2022; Smyser et al., 2020). It is plausible that the genetic composition of individuals in other populations with varying proportions of wild boar, heritage breeds, or highly fecund improved commercial breeds of domestic pig ancestry (as has been asserted for emergent invasive populations in Canada; Aschim & Brook, 2019) could contribute to the variance in sex ratio. Therefore, investigation of other localized populations' ancestry is needed to determine the genetic composition and subsequently include the ancestry into modeling frameworks to better understand potential drivers of litter size and sex ratio.

Our results suggest additional attributes contribute to the variance in fetal sex ratios that were not accounted for in our models. Our environmental variable, food availability (measured as the amount of extraneous fat), was included in the competitive models influencing sex ratio. However, as none of the models were better than the null model, we do not have a clear understanding of food availability on fetal sex ratio. While food resources are important for reproduction, it is not clear how food abundance and diet influence sex ratio for species that have multiple offspring per reproductive event (Rosenfeld et al., 2003; Rosenfeld & Roberts, 2004). Wild pigs exhibit a high degree of behavioral plasticity, exploiting environmental fluctuations such as increased food resources (i.e., high mast season) by increasing litter size (Frauendorf et al., 2016; Massei et al., 1996). By taking advantage of pulses of high‐quality resources, females may be able to bridge the energetic gap necessary to produce more offspring and, in concept, successfully wean more piglets to maximize lifetime fitness. Another abiotic factor that we did not account for but could influence fetal sex ratio was the sex ratio of the wild pig population on the SRS. Clancey and Byers (2016) found that from demographic stochasticity over time, the local pronghorn (*Antilocarpa americana*) population showed a trend that when there were few males, the fawn cohort in the subsequent spring was significantly male‐biased. While management of wild pig populations, including the SRS population, is largely unbiased for sex, demographic fluctuations in localized populations may influence litter sex ratio.

For other ungulates, maternal age influences offspring sex ratio (Côté & Festa‐Bianchet, 2001; Saltz, 2001; Thomas et al., 1989; but see Hamel et al., 2016, where the trend disappeared with a larger dataset which can be a limiting factor in wildlife studies) and it is found to influence reproductive ecology in wild pigs such that older females contribute most to population growth (Chinn et al., 2022). Older females are usually larger, which correlates with larger energy stores and the ability to deposit fat reserves, relative to resource availability (Mayer, Smyser, et al., 2020). Thus, older females may be able to invest more energy into the gestation of larger litters (as seen in this study), more male offspring, and milk production for more and larger male piglets. While it was not influential in our sex ratio model framework, and with the constraint of small sample sizes across age classes, maternal age may be an important parameter affecting sex ratio and warrants further investigation.

For polytocous species, Williams (1979) predicted mothers maximize fitness by increasing allocation toward litter production, in relation to their condition. Wild pigs exhibited ~2% higher maternal expenditure per individual male offspring compared to females during pregnancy, which increased toward the end of gestation and would likely continue through lactation. Wild pigs produce multiple offspring per litter and the additive cost of producing multiple male offspring may result in a significant difference between the sexes as the fetal sex ratio increases and becomes male‐biased. We found that the maternal quality was a good predictor of female allocation to litter production such that allocation increased as the percent of maternal parent wild boar ancestry and condition increased. Sex allocation studies of other polytocous mammals are variable in support of TWM and the Williams Model or support these predictions only under certain environmental conditions because the trade‐offs between sex ratio and litter size complicate predictions of offspring sex in relation to maternal quality for polytocous species (e.g., marmot [*Marmota flaviventris*]: Armitage, 1987; mice [*Mus musculus*]: Dušek et al., 2011; squirrel [*Urocitellus richardsonii*]: Gedir & Michener, 2014). Further, for mammals, allocation to offspring continues after birth and is most expensive through lactation (Hanwell & Peaker, 1977). Thus, the cost differential between male and female offspring is likely to be greater than our estimates of fetal mass alone, especially during lactation when the energy required to feed a male would likely exceed the extra allocation to a male compared to a female during gestation. When we addressed this trade‐off, we found that females preferentially adjusted litter size and not sex ratio in relation to their condition, and therefore, under these specifications, the Williams model was not supported.

In this study, we found that it may be optimal for individuals to adjust litter size to increase reproductive success, rather than sex ratio. Polytocous species may employ a combination of sex allocation strategies: maximizing sex ratio in terms of the optimal litter size (Myers, 1978), adjusting litter size and sex ratio to optimize fitness (Williams, 1979), and a “maternal coping strategy,” where females in poor condition will maximize the number of males in the litter at the expense of their own condition (i.e., will increase the proportion of males in the litter with increased loss of maternal mass, Dušek et al., 2011). Since mechanisms of primary sex ratio adjustment are uncertain, true relationships may only be applicable in relation to measurements of maternal quality prior to conception, however, the relative role of maternal attributes pre‐ and postconception remains unclear (Sheldon & West, 2004). For in situ studies of wild animals, most measurements are limited to pregnant individuals, with little or no prior knowledge of previous conditions. Factors affecting primary and secondary sex ratio rates could differ and should be considered during the experimental design phase of a study to ensure relevant parameters are tested.

## AUTHOR CONTRIBUTIONS

**Sarah M. Chinn:** Conceptualization (equal); data curation (equal); formal analysis (equal); investigation (equal); methodology (equal); project administration (equal); validation (equal); visualization (equal); writing – original draft (equal); writing – review and editing (equal). **Timothy Smyser:** Formal analysis (equal); investigation (equal); methodology (equal); writing – review and editing (equal). **James C. Beasley:** Conceptualization (equal); funding acquisition (equal); investigation (equal); methodology (equal); project administration (equal); resources (equal); supervision (equal); writing – original draft (equal); writing – review and editing (equal).

## CONFLICT OF INTEREST STATEMENT

The authors declare that they have no known competing financial interests or personal relationships that could have appeared to influence the work reported in this manuscript.

## DISCLAIMER

The manuscript was prepared as an account of work sponsored by an agency of the US government. Neither the US government nor any agency thereof, nor any of its employees, makes any warranty, express, or implied or assumes any legal liability or responsibility for the accuracy, completeness, or usefulness of any information disclosed or represents that its use does not infringe privately owned rights. Reference herein to any specific commercial product, process, or service by trade name, trademark, manufacturer, or otherwise does not constitute or imply its endorsement, recommendation, or favoring by the US government or any agency thereof. The views and opinions of the authors expressed herein do not necessarily state or reflect those of the US government or any agency thereof.

## Data Availability

Data are accessible on the Dryad Digital Repository: https://doi.org/10.5061/dryad.pzgmsbcrz.
